# Socioeconomic inequalities in pandemic-induced psychosocial stress in different life domains among the working-age population

**DOI:** 10.1186/s12889-024-18874-3

**Published:** 2024-05-28

**Authors:** Florian Beese, Benjamin Wachtler, Markus M. Grabka, Miriam Blume, Christina Kersjes, Robert Gutu, Elvira Mauz, Jens Hoebel

**Affiliations:** 1 Department of Epidemiology and Health Monitoring, Robert Koch Institute, Berlin, Germany; 2 Socio-Economic Panel, German Institute for Economic Research, Berlin, Germany; 3 Institute of Medical Sociology, Centre for Health and Society, Medical Faculty, Heinrich-Heine-University, Düsseldorf, Germany

**Keywords:** Socioeconomic position, Psychosocial stress, COVID-19 pandemic, RKI-SOEP-2, Life domains

## Abstract

**Background:**

Psychosocial stress is considered a risk factor for physical and mental ill-health. Evidence on socioeconomic inequalities with regard to the psychosocial consequences of the COVID-19 pandemic in Germany is still limited. We aimed to investigate how pandemic-induced psychosocial stress (PIPS) in different life domains differed between socioeconomic groups.

**Methods:**

Data came from the German Corona-Monitoring nationwide study – wave 2 (RKI-SOEP-2, November 2021–February 2022). PIPS was assessed using 4-point Likert scales with reference to the following life domains: family, partnership, own financial situation, psychological well-being, leisure activity, social life and work/school situation. Responses were dichotomised into “not stressed/slightly stressed/rather stressed” (0) versus “highly stressed” (1). The sample was restricted to the working-age population in Germany (age = 18–67 years, *n* = 8,402). Prevalence estimates of high PIPS were calculated by sex, age, education and income. Adjusted prevalence ratios (PRs) were estimated using Poisson regression to investigate the association between education/income and PIPS; high education and income were the reference groups.

**Results:**

The highest stress levels were reported in the domains social life and leisure activity. Women and younger participants reported high stress levels more frequently. The highest inequalities were found regarding people’s own financial situation, and PIPS was higher in low vs. high income groups (PR 5.54, 95% CI 3.61–8.52). Inequalities were also found regarding partnerships with higher PIPS in low vs. high education groups (PR 1.68, 95% CI 1.13–2.49) – and psychological well-being with higher PIPS in low vs. high income groups (PR 1.52, 95% CI 1.14–2.04).

**Conclusion:**

Socioeconomic inequalities in PIPS were found for different life domains. Generally, psychosocial support and preventive interventions to help people cope with stress in a pandemic context should be target-group-specific, addressing the particular needs and circumstances of certain socioeconomic groups.

**Supplementary Information:**

The online version contains supplementary material available at 10.1186/s12889-024-18874-3.

## Introduction

After the COVID-19 pandemic was declared by the World Health Organization in March 2020 [1], the world faced an immense health crisis. Besides high infection rates with SARS-CoV-2 during several pandemic infection waves, numerous people were hospitalised, suffered from long-term health consequences or died from COVID-19 [2]. Social epidemiological research uncovered the socially unequal distribution of these direct health consequences of COVID-19 over the course of the pandemic and showed that groups with a lower socioeconomic position were more likely to become infected with SARS-CoV-2, get hospitalised or to die due to COVID-19 [3].

To reduce the spread of SARS-CoV-2, non-pharmaceutical interventions (NPIs) – such as physical distancing, quarantine measures for infected individuals, school closures and visiting restrictions for relatives in hospitals and nursing facilities – were introduced in the majority of countries worldwide. These measures had immense consequences for individuals and societies [4]. NPIs were implemented to reduce transmissions of SARS-CoV-2, but may have had side effects on individuals’ health, e.g. as a result of the profound change in daily routines and their health impacts. On the one hand, social restrictions may have led to stress in different life domains such as family life (e.g. to organising work and childcare at home) [5]. On the other hand, these restrictions may have had a negative impact on individual coping strategies against stress such as leisure activities or support via social networks [6]. Furthermore, the closures of numerous establishments and economic uncertainty may have led to financial worries due to (potential) loss of income or employment [4].

There are many theories and models to describe stress. One influential model is the Transactional Model of Stress and Coping from Lazarus and Folkman [7]. According to this model, a situation is perceived as stressful depending on the interpretation of the stressor and the evaluation of one’s resources or coping strategies. Limited socioeconomic resources may lead to a higher susceptibility and vulnerability to stressors in different life domains and to coping strategies that are less conducive to health [8–11]. During the COVID-19 pandemic, individuals with higher socioeconomic position might see the pandemic as a manageable challenge and engage in problem-focused coping, such as improving their home for remote work, leading to more effective handling of emerging changes in daily routines. In contrast, those facing socioeconomic disadvantages may view the pandemic more as a threat due to limited resources, financial instability, and the necessity to work in high-risk environments without the option of remote work. These conditions can exacerbate the perception of vulnerability and lack of control, steering them towards more maladaptive coping mechanisms, such as denial, which might offer immediate psychological relief but can lead to less adaptive outcomes in the long-term [12]. Moreover, the chronic stress associated with socioeconomic disadvantages can erode coping resources over time, making it more challenging to employ adaptive coping strategies [13].

Investigating socioeconomic inequalities in psychosocial stress is important, since stress is a risk factor for a variety of health issues due to its impact on psychological and biological processes. It affects health in different ways, particularly via neuroendocrinological pathways such as the hypothalamic-pituitary-adrenocortical axis, but also as a result of changes in certain behaviours [14]. Accumulative stress over the life course, relating to the concept of allostatic load [15], is considered as major contributor to the emergence and reproduction of health inequalities [16]. Empirical findings on pandemic-induced stress during the COVID-19 pandemic are generally still limited, especially for the general population, and evidence on socioeconomic inequalities relating to such stress is still scarce. However, such knowledge can provide useful insights for targeted supportive interventions and prevention during pandemics. Hence, the objective of this paper was to investigate socioeconomic inequalities in pandemic-induced psychosocial stress (PIPS) in different life domains among the general population in Germany during the COVID-19 pandemic.

## Methods

### Study design

The ‘Corona Monitoring Nationwide (RKI-SOEP-2)’ study – a cooperative project of the Robert Koch Institute and the Socio-Economic Panel (SOEP) at the German Institute for Economic Research with the Institute for Employment Research and the Research Centre of the German Federal Office for Migration and Refugees – was hosted in the SOEP. The SOEP is a German nationwide dynamic cohort based on population-based random samples [17], which allows representative statements to be made about individuals living in private households across Germany. Data were collected between November 2021 and February 2022. All SOEP households in the gross sample were invited to participate in the study. An invitation package was sent to each target person containing both an individual invitation and study materials (i.e. questionnaire, blood self-sampling kit for capillary blood). Respondents could fill in the self-administered questionnaire either in paper form or online. The questionnaire covered topics such as experienced SARS-CoV-2 infections, COVID-19 vaccination status and willingness to be vaccinated, psychosocial stress, health status, and health behaviour. A more detailed description of the RKI-SOEP-2 study can be found elsewhere [18]. We restricted our analysis to the working-age population between the ages of 18 and 67 assuming that this population may have been particularly exposed to intertwining and accumulating stressors, such as personal development, balancing work and family responsibilities, remote work challenges, or home schooling during the pandemic [19]. Furthermore, comparability with groups such as retirees might be limited, as they often have different financial and social support structures compared to the working-age population.

### Pandemic-induced psychosocial stress

PIPS was assessed with the survey question “Overall, how much have you been stressed due to the pandemic? With regard to…”, followed by a list of life domains: family, partnership, own financial situation, work/school, social life, psychological well-being and leisure activities. Participants could answer the question separately for each life domain using a 4-point Likert scale (“not stressed”, “slightly stressed”, “rather stressed”, “highly stressed”). If respondents wanted to indicate that one of the life domains was not relevant to them (e.g. questions about partnership for singles), the additional response option “does not apply” was possible. In the analysis, “does not apply” was treated as a missing value instead of assigning it to one of the other response categories, in order to avoid making assumptions about the answering behaviour of the participants. PIPS was dichotomised into “not stressed/slightly stressed/rather stressed” (0) vs. “highly stressed” (1). We conducted a sensitivity analysis by defining “does not apply” as “not stressed”. For an additional sensitivity analysis, we dichotomised PIPS into “not stressed/slightly stressed” vs. “rather stressed/highly stressed”. We also performed a correlation analysis using Spearman’s rank correlation for ordinal variables to assess the correlations of PIPS between the different life domains (see table supp. 1 in Additional file 1).

### Socioeconomic position

Socioeconomic position was assessed using education and income. Education was measured by the International Standard Classification of Education (ISCED) version 2011 [20], and categorised as low (lower secondary education or below), medium (upper secondary or post-secondary education) and high (tertiary education). Income was operationalised by the current equivalised net household income using the square-root method [21]. The equivalised household income was further categorised as low (quintile 1), medium (quintile 2–4) and high (quintile 5).

### Statistical analysis

We estimated prevalence rates with 95%-confidence intervals (95% CI) of PIPS by age, sex, education and income. Furthermore, we fitted multivariate Poisson regression models to estimate adjusted prevalence ratios (PR) and 95% CI with household-clustered standard errors to investigate the association between socioeconomic position (with high education and high income as reference groups) and PIPS. Poisson regression was used because the prevalence estimates of the outcomes are high and, in this case, logistic regression might result in overestimated coefficients, while Poisson regression allows the direct estimation of PR [22].

For the multivariate analyses we adjusted for age and sex, employment status, household composition (differentiating for the presence of children under the age of 16), and the frequency of working from home. PRs by income were additionally adjusted for education since educational attainment is commonly causally anterior to income and thus a potential confounder of the income-outcome associations. We ran the models for each combination of domain-specific PIPS and socioeconomic indicator separately leading to a total number of 14 models in the main analysis. As an additional analysis, we further conducted the multivariate analyses stratified by sex.

All analyses were conducted using weighting factors to adjust for systematic non-response. The weights result from non-response modelling at the household and individual level and adjust the sample to match the German micro census by age, sex, citizenship, federal state, household type/size and owner-occupied housing [23]. All analyses were conducted using R version 4.3.0 [24].

## Results

**Fig. 1:**
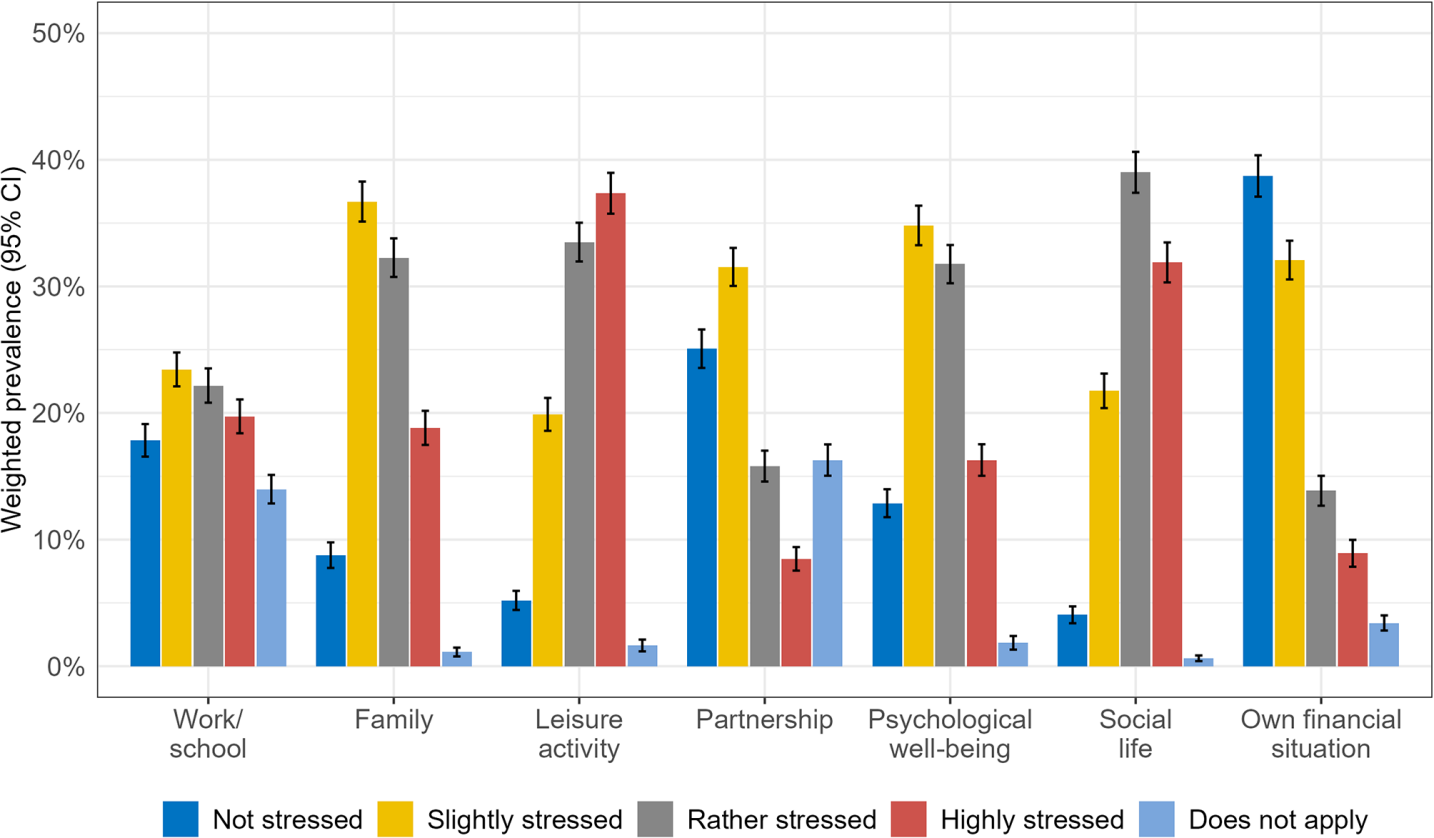
Life-domain-specific prevalence estimates of pandemic-induced psychosocial stress

In total, 8,402 working-age participants were included in the analyses. Table 1 shows the sample characteristics. The PIPS response option “does not apply” was most prevalent in the life domains work/school and partnership (see Fig. 1). In the correlation analysis of the outcome variables, partnership stress and family stress showed the strongest correlation (0.43), followed by the correlation between social-life stress and leisure-activity stress (0.41). We found the lowest correlation (0.14) between partnership stress and work/school stress as well as between partnership stress and leisure-activity stress (see table supp. 1 in Additional file 1).

**Table 1:** Characteristics of the study population (*n* = 8,402)

		*n*		%*
Sex	Female	4,627		49.5
	Male	3,775		50.5
	Missing	-		-
Age group	18–29 years	1,319		20.4
	30–49 years	3,092		39.2
	50–67 years	3,991		40.4
	Missing	-		-
Education	Low	800		10.7
	Medium	3,904		52.6
	High	3,252		36.7
	Missing	446		-
Income	Low	1,224		20.0
	Medium	4,566		60.0
	High	2,014		20.0
	Missing	598		-
Employment	Full-time	3,804		51.6
	Part-time	1,647		18.4
	Marginal/irregular	485		7.3
	Not employed	1,576		22.6
	Missing	890		-
Household composition	Single	1,161		24.7
	Multi-person without children under 16 years	2,983		31.2
	Multi-person with children under 16 years	3,942		44.2
	Missing	316		-
Working from home	Daily	967		12.8
	Several times/week	1,200		13.8
	Every two weeks or less	700		8.3
	No working from home	3,282		43.2
	Not employed	1,576		21.9
	Missing	677		-
Family stress	Yes	1,645		19.5
	No	6,456		80.5
	Missing	301		-
Partnership stress	Yes	708		10.5
	No	6,380		89.5
	Missing	1,314		-
Own financial situation stress	Yes	655		9.5
	No	7,220		90.5
	Missing	527		-
Social-life stress	Yes	2,618		33.0
	No	5,481		67.0
	Missing	303		-
Work/school stress	Yes	1,224		22.3
	No	4,367		77.7
	Missing	2,811		-
Psychosocial well-being stress	Yes	1,364		17.0
	No	6,693		83.0
	Missing	345		-
Leisure-activity stress	Yes	3,128		38.9
	No	4,944		61.1
	Missing	330		-

n = unweighted number of participants; * weighted %; % figures do not necessarily add up to 100% due to rounded values

### Prevalence of pandemic-induced psychosocial stress

Figure 1 shows the life-domain-specific prevalence estimates of PIPS in its ordinal variable version. In general, own financial situation was mostly perceived as being not stressing (38.7%, 95% CI 37.1–40.4%) while leisure time was mostly perceived as being highly stressful (37.4%, 95% CI 35.7–39.0%). Family, partnership, psychological well-being and work/school were mostly perceived as being slightly stressful (family 36.7%, 95% CI 35.1–38.3%; partnership 31.5%, 95% CI 30.0–33.0%; psychological well-being 34.8%, 95% CI 33.3–36.4%; work/school 23.4%, 95% CI 22.1–24.8%). Social life was mostly perceived as being rather stressful (39.0%, 95% CI 37.4–40.6%).

**Fig. 2:**
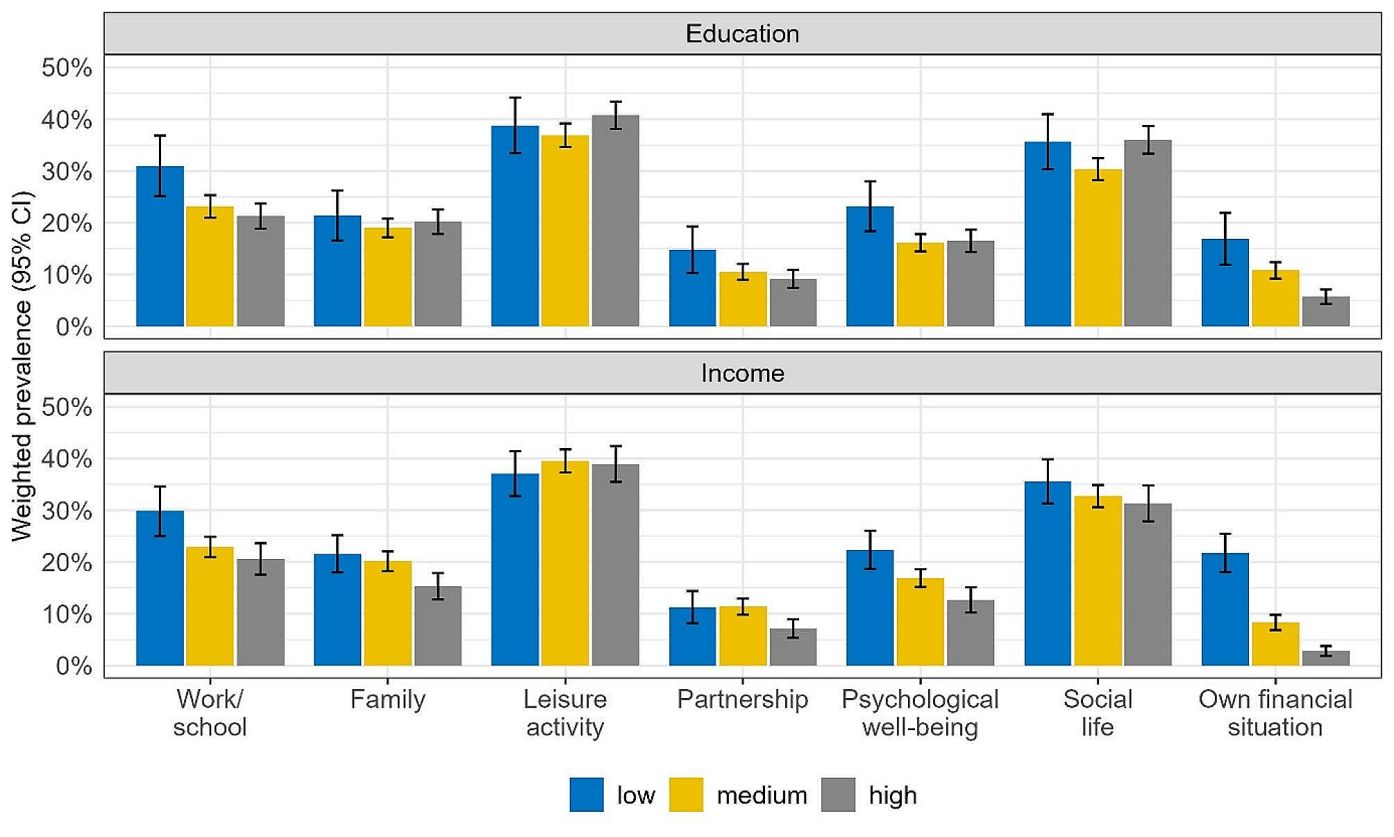
Life domain-specific prevalence estimates of high pandemic-induced psychosocial stress by education and income

Figure 2 shows the life-domain-specific prevalence estimates of high PIPS in its dichotomised version according to education and income (the exact estimates and 95% CIs can be found in table supp. 2 in Additional file 1). In general, the highest prevalence of high PIPS was found in the life domains leisure activity and social life with no profound differences by education or income. The lowest prevalence of high PIPS was found regarding partnership and own financial situation, with a social gradient in the latter to the disadvantage of groups with a lower education or income. Groups with a lower education and income further reported high PIPS regarding work/school and psychological well-being more frequently. The prevalence of high PIPS by sex and age can be found in the supplementary materials (see figure supp. 1 and 2 in Additional file 1). PIPS increased with younger age and for females in almost all domains except own financial situation. In addition, younger participants reported lower PIPS in the domain family compared to the other age groups.

### Multivariate analyses

**Fig. 3:**
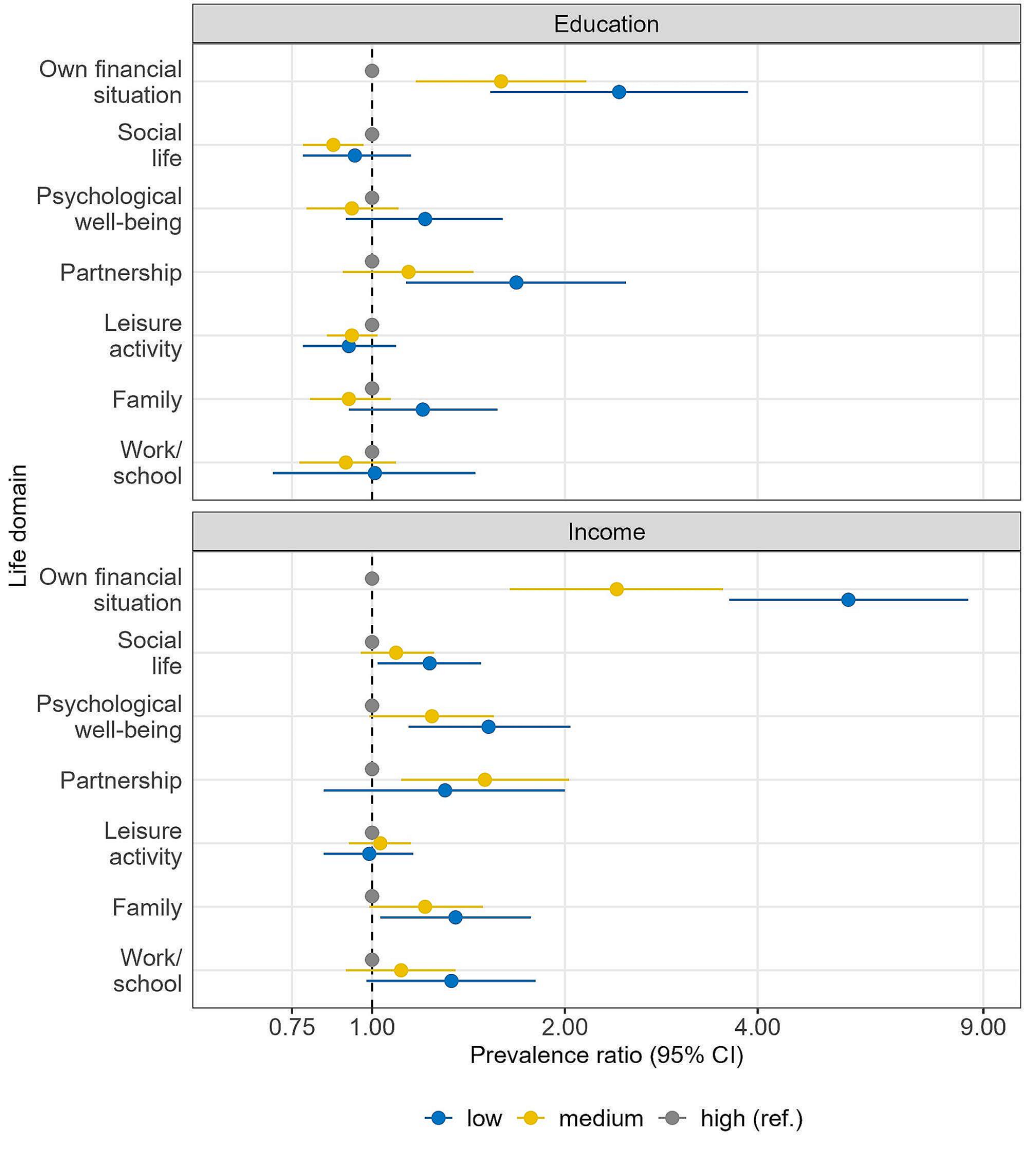
Adjusted prevalence ratios and 95%-confidence intervals (95% CI) for high pandemic-induced psychosocial stress by education and income (adjusted for age, sex, employment status, frequency of working from home and household composition)

Figure 3 shows the multiple-adjusted PRs with 95% CI of high PIPS by education and income. The exact PR, 95% CI, and p-values can be found in table supp. 3 in Additional file 1. Low education was associated with a higher prevalence of being highly stressed in one’s partnership (PR 1.68, 95% CI 1.13–2.49) and own financial situation (PR 2.43, 95% CI 1.53–3.86). Medium education was associated with a higher prevalence of being highly stressed regarding one’s own financial situation as well (PR 1.59, 95% CI 1.17–2.16), and with a lower prevalence of being highly stressed in the domain social life (PR 0.87, 95% CI 0.78–0.97) compared to the reference of high education. Groups with a low income had a higher prevalence of being highly stressed in their family life (PR 1.35, 95% CI 1.03–1.77), social life (PR 1.23, 95% CI 1.02–1.48), psychological well-being (PR 1.52, 95% CI 1.14–2.04) and own financial situation (PR 5.54, 95% CI 3.61–8.52). Groups with medium income had a higher prevalence of feeling highly stressed in their partnership (PR 1.50, 95% CI 1.11–2.03) and own financial situation (PR 2.41, 95% CI 1.64–3.53) as compared to those with high incomes.

The sex-stratified multivariate analysis showed that especially women were affected by socioeconomic inequalities in PIPS. Significant inequalities were found regarding family, partnership, own financial situation, social life, psychological well-being and leisure activity (table supp. 4a in Additional file 1). Men only showed significant inequalities regarding the own financial situation (table supp. 4b in Additional file 1).

Our sensitivity analysis by setting the answer “does not apply” to “not stressed” showed marginal changes in the coefficients while none of the associations altered substantially (table supp. 5 in Additional file 1). When the outcome was defined as “rather/highly stressed”, some results deviated from the results on “highly stressed”, mostly changing to no associations (table supp. 6 in Additional file 1). However, while the associations between low or medium education/income and PIPS regarding own financial situation remained the strongest across the life domains, we found that groups with a lower education or income showed a lower prevalence of being rather/highly stressed regarding leisure activity compared to groups with high education (PR 0.91, 95% CI 0.83–0.99) or income (PR 0.90, 95% CI 0.83–0.98). A similar association was found regarding social-life stress and lower education (PR 0.91, 95% CI 0.84–0.99). The prevalence of being rather/highly stressed relating to psychological well-being remained higher among groups with lower income (PR 1.17, 95% CI 1.03–1.33).

## Discussion

### Summary of results

This is the first study on socioeconomic inequalities with regard to domain-specific psychosocial stress induced by the COVID-19 pandemic among Germany’s working-age population. In general, the highest levels of PIPS overall were found in the life domains leisure activity and social life. Across several life domains, our analysis shows inequalities in PIPS by different socioeconomic indicators. Socioeconomic inequalities were largest in PIPS regarding one’s own financial situation, and the highest prevalence was among those with a lower education or income. Furthermore, women with a lower education or income showed a higher prevalence of being stressed in other domains such as family, partnership or psychological well-being during the pandemic.

### Family and partnership

Our results indicate that participants with lower income had a higher prevalence of perceiving family stress compared to participants with higher income, which is in line with findings from the United States [25]. One possible explanation here is the increased financial strain for those with lower income, which was also found in our analyses and might have led to additional worries and frictions in the family. Furthermore, an increase in domestic conflicts and violence during the pandemic might be a relevant factor here. A narrative review did not find a general increase for Germany but argues that changes may have occurred in subgroups [26]. Risk factors for physical partnership violence at the beginning of the pandemic were home quarantine, financial worries and young children in the household [27]. Increased partnership stress was found in groups with low education and medium income. Our correlation analysis showed that the family-partnership correlation was among the highest correlations, which might in part explain the similar results. Other underlying mechanisms might play a role here, such as different household compositions or other constellations, which were outside the scope in our analysis. Research focusing on different family constellations and the role of partnership in this context might contribute to a better understanding of these mechanisms.

### Own financial situation

While the prevalence of pandemic-induced stress due to a person’s own financial situation was generally low compared to PIPS in other domains, socioeconomic inequalities were found to be largest in this domain to the disadvantage of groups with low education or income. Participants with low education or low income may be at higher risk of loss of income, job loss or stressful employment conditions in the pandemic context [28]. Financial stress is considered a risk factor for adverse mental health. One study from Thailand found that the risk of depressive symptoms or anxiety doubled among those who perceived financial stress in the pandemic [29], and this is also supported by other studies [30–33]. Although the absolute level of PIPS regarding the people’s own financial situation was low compared to PIPS in other domains, our findings on socioeconomic inequalities in this respect can help identify target groups that could particularly benefit from financial support or regulations protecting employees from job and income loss in pandemic situations in order to prevent financial stress.

### Psychological well-being

The results on psychological well-being show that those with a low income experienced higher stress levels in this domain than those with high incomes. These results are in line with other international research, which showed higher levels of psychological distress in people with lower incomes [34, 35]. One influencing factor might be fear of COVID-19 itself. For instance, people with a low income were found to perceive a SARS-CoV-2 infection as more dangerous [36]. A meta-analysis further found that fear of COVID-19 contributed moderately to greatly to stress, as well as to other kinds of mental health difficulties, such as depression, anxiety, sleep problems and impaired well-being [37]. We found no association with education, which is in line with research suggesting that educational inequalities in psychological well-being and stress narrowed during the early pandemic [38, 39].

### Social life

Despite some inequalities in terms of a higher risk of perceiving stress among groups with low incomes, we found no socioeconomic inequalities for the domain social life. However, the stress levels in social life were among the highest in our analyses. This reflects a generally high burden of pandemic-induced stress in this domain among Germany’s working-age population, which might also have an influence on the social support capabilities to cope with stress in other domains. The high overall prevalence is also plausible due to the facts that nearly everybody was affected by contact restrictions during the pandemic, such as limitations on or prohibitions of social meetings or even complete lockdowns with stay-at-home orders.

### Work/school

A comparatively lower prevalence level was found for PIPS in the work or school domain, which is in line with other research reports from Germany [40]. While the crude prevalence in this domain was higher among those with low education or income, we did not find any multivariate association of high stress in this domain. These are surprising results as groups with lower socioeconomic status are more frequently employed in essential jobs [41]. They were prompted to continue their work and were exposed to different stressors, such as higher work loads or higher mobility, when the majority of the population was urged to reduce their mobility to reduce the spread of SARS-CoV-2. It was found that people with low incomes were less likely to have the opportunity to work from home/remotely [31, 42]. At the same time, this meant that these people could not choose their degree of exposure to the virus, but had to continue working, e.g. in contact with other people, which could therefore lead to stress. One explanation why we found no association could be that also people who were able to work from home also perceived the change of the work situation as stressful as this mostly has required dealing with work, household duties or care work at the same time.

### Leisure activity

The results for PIPS in the domain leisure activity might seem surprising. However, the reverse association compared to the other results (lower education less associated with stress) may be a result of the introduction and promotion of short-time work in Germany due to the COVID-19 pandemic. There is evidence from the pandemic that groups with a lower education were more frequently prompted to reduce their working hours [43] and therefore had more time for leisure activities compared to those who were able to work from home (which is more frequently actionable among groups with a higher education). Another explanation could be that the NPIs also had positively perceived side effects. These may include the deepening of friendship and intimate relationships, the perception of more free time, and the opportunity to try out new activities [44]. These positive side effects are likely to be particularly relevant for people with low incomes or low levels of education, as they usually report a low level of control. Furthermore, those with a higher education or income may have suffered more from the economic shock with a greater impact on the lifestyle for those in better socioeconomic circumstances [45].

In general, our results have to be discussed within the context of the working age. Working age represents a critical period for economic productivity, social contribution, and personal development [19]. During the pandemic, this population faced unique pressures, including the balancing of employment and family responsibilities, adaptation to remote work or unemployment, and the navigation of social isolation measures. These challenges were further exacerbated by pre-existing socioeconomic inequalities, as our findings demonstrate.

### Strengths and limitations

To our knowledge, this is the first study investigating socioeconomic inequalities in PIPS in different life domains that allows for a differentiated investigation of this topic. Furthermore, combining the RKI-SOEP wave 2 data with the SOEP panel data enabled us to analyse socioeconomic variables and to include more potentially relevant variables such as employment status or household composition.

Despite these strengths, several limitations should be noted. Although the underlying survey question on stress (see methods) refers to the pandemic context, it is not clear which period of the pandemic was considered by the participants in their responses. Because the survey was conducted from late 2021 until early 2022 participants were perhaps referring to a period when NPIs were highly present or to a period when the daily life was close to normal. This may have had an influence on the response behaviour, and a decomposition of the influence of certain NPIs on domain-specific psychosocial stress was therefore not possible. A lot of previous research investigated the association between psychosocial stress and several restrictions imposed during the pandemic. However, because of the survey question, our results cannot discriminate between different measures but have to be interpreted within the broader context of the pandemic as a whole. Moreover, it is not fully clear how respondents perceived the question about stress in our survey, as it does not utilize an established instrument to measure stress, such as Cohen’s Perceived Stress Scale [46]. This limitation might have resulted in lower validity in the measurement of pandemic-induced stress in our study compared to other more general scales. In addition, we cannot rule out the possibility that the survey responses may include some level of perceived stress not directly attributable to the pandemic, even though the question explicitly asked about pandemic-induced psychosocial stress. For instance, participants who perceive high levels of stress at work, independent of the pandemic, might be more likely to report high stress levels in our survey. Regarding the surveyed domains, it is a shortcoming of our study that stressors such as own illness or illness of close relatives were not assessed. There is evidence that disease-related stressors contribute significantly to perceived stress [47]. These stressors are likely magnified during a pandemic due to heightened health concerns and potential disruptions in social and healthcare services. Moreover, disease-related stressors might have been unevenly distributed across socioeconomic groups, which would have been another relevant aspect to consider in our analyses and pertinent to public health. A further limitation is that the answer “does not apply” might have been misunderstood and interpreted as “not stressed”. For that reason, we conducted sensitivity analyses, which showed that the results predominantly remained robust when different assumptions were applied. A further limitation might be the conceptual interpretation of some of the life-domains. For instance, some participants may have interpreted the domain family as their family within the household (e.g. partner and children) while other participants may have thought of their relatives (e.g. parents, grandparents, aunt, etc.) they were unable to visit due to restrictions. The analysed life domains therefore only draw a general picture of stress levels in these domains. Further, more in-depth research is needed to investigate the underlying mechanisms. For instance, being employed in a specific sector or professional field, such as healthcare, probably had a different impact on PIPS compared to other jobs. Furthermore, more differentiated aspects of the employment situation such as being self-employed versus being employed can be assumed to have affected PIPS, especially regarding the own financial situation. These aspects were out of the scope of our analyses but were already addressed elsewhere [48]. It also must be borne in mind that cross-sectional observational data were analysed, and these do not allow causal inferences about the associations found.

## Conclusion

The results indicate socioeconomic inequalities in PIPS among Germany’s working-age population. Indirect psychosocial consequences of the pandemic may in some cases have been distributed differently across socioeconomic groups, depending on life-domain-specific particularities. More in-depth research is needed to evaluate and contextualise these results in more detail and to explore the underlying mechanisms. Generally, psychosocial support and preventive interventions to help people cope with stress in a pandemic context should be target-group-specific by addressing the particular needs and circumstances of certain socioeconomic groups. Regulations to prevent disadvantaged populations from adverse consequences from a pandemic situation, such as loss of income or job loss as a result of pandemic lockdowns, are relevant to public health in the pandemic context. These might reduce perceived stress and the associated indirect health consequences of a pandemic.

### Electronic supplementary material

Below is the link to the electronic supplementary material.

Supplementary Material 1

## Data Availability

The data cannot be made publicly available because informed consent from participants did not cover the public deposition of data. However, the data underlying the analysis in this article is archived in the SOEP Research Data Centre (https://www.diw.de/en/diw_01.c.601584.en/data_access.html) in Berlin and can be accessed on site upon reasonable request.

## Abbreviations

CI: Confidence interval
COVID-19: Coronavirus Disease 2019
NPI: Non-pharmaceutical intervention
PIPS: Pandemic-induced psychosocial stress
PR: Prevalence ratio
RKI-SOEP-2: ‘Corona Monitoring Nationwide’ study
SARS-CoV-2: Severe acute respiratory syndrome coronavirus type 2
SOEP: Socio-Economic Panel
