# Supplementary material for: Socioeconomic inequalities in pandemic-induced psychosocial stress in different life domains among the working-age population
Source: BMC Public Health. 2024 May 28;24:1421. doi: 10.1186/s12889-024-18874-3 (PMC11131271; doi:10.1186/s12889-024-18874-3)
Supplement: Supplementary file 1 — Supplementary Material 1 [file 12889_2024_18874_MOESM1_ESM.docx]

Additional file 1

**Socioeconomic inequalities in pandemic-induced psychosocial stress in different life domains among the working-age population**

Florian Beese^1*^, Benjamin Wachtler^1^, Markus M. Grabka^2^, Miriam Blume^1,3^, Christina Kersjes^1^, Robert Gutu^3^, Elvira Mauz^1^, Jens Hoebel^1^

^1^Department of Epidemiology and Health Monitoring, Robert Koch Institute, Berlin, Germany

^2^Socio-Economic Panel, German Institute for Economic Research, Berlin, Germany

^3^Institute of Medical Sociology, Centre for Health and Society, Medical Faculty, Heinrich-Heine-University, Düsseldorf, Germany

* Corresponding author

**Content:**

**Table supp. 1** Correlation between life domain-specific stress variables.

**Table supp. 2** Weighted prevalence estimates (%) and 95% confidence intervals (95% CI) for high pandemic-induced psychosocial stress by education and income.

**Figure supp. 1** Life domain-specific prevalence estimates of pandemic-induced psychosocial stress (highly stressed) by sex.

**Figure supp. 2** Life domain-specific prevalence estimates of pandemic-induced psychosocial stress (highly stressed) by age.

**Table supp. 3** Adjusted prevalence ratios and 95%-confidence intervals (95% CI) for high pandemic-induced psychosocial stress by education and income (with high education and income as reference categories).

**Table supp. 4a** Adjusted prevalence ratios and 95%-confidence intervals (95% CI) for high pandemic-induced psychosocial stress by education and income (with high education and income as reference categories) among women.

**Table supp. 4b** Adjusted prevalence ratios and 95%-confidence intervals (95% CI) for high pandemic-induced psychosocial stress by education and income (with high education and income as reference categories) among men.

**Table supp. 5** Adjusted prevalence ratios and 95%-confidence intervals (95% CI) for high pandemic-induced psychosocial stress by education and income (with high education and income as reference categories) with "does not apply" operationalized as not stressed.

**Table supp. 6** Adjusted prevalence ratios and 95%-confidence intervals (95% CI) for pandemic-induced psychosocial stress (“rather stressed”/”highly stressed”) by education and income (with high education and income as reference categories).

**Table supp. 1** Correlation between life domain-specific stress variables

|  | (1) | (2) | (3) | (4) | (5) | (6) | (7) |
| --- | --- | --- | --- | --- | --- | --- | --- |
| Family (1) | 1 |  |  |  |  |  |  |
| Partnership (2) | 0.43 | 1 |  |  |  |  |  |
| Own financial situation (3) | 0.18 | 0.17 | 1 |  |  |  |  |
| Social life (4) | 0.29 | 0.19 | 0.16 | 1 |  |  |  |
| Work/school (5) | 0.18 | 0.14 | 0.26 | 0.26 | 1 |  |  |
| Psychological well-being (6) | 0.32 | 0.28 | 0.22 | 0.35 | 0.25 | 1 |  |
| Leisure activity (7) | 0.22 | 0.14 | 0.13 | 0.41 | 0.2 | 0.32 | 1 |

**Table supp. 2** Weighted prevalence estimates (%) and 95% confidence intervals (95% CI) for high pandemic-induced psychosocial stress by education and income.

|  |  | **Education** | | |
| --- | --- | --- | --- | --- |
|  |  | **low** | **medium** | **high** |
|  |  | % (95% CI) | % (95% CI) | % (95% CI) |
| Family |  | 21.4 (16.5–26.2) | 19.0 (17.2–20.8) | 20.2 (17.8–22.5) |
| Partnership |  | 14.8 (10.3–19.3) | 10.5 (9.0–12.0) | 9.1 (7.4–10.8) |
| Own financial situation |  | 16.9 (11.9–21.9) | 10.8 (9.2–12.4) | 5.7 (4.3–7.1) |
| Social life |  | 35.6 (30.3–40.9) | 30.3 (28.2–32.5) | 36.0 (33.3–38.7) |
| Work/ school |  | 31.0 (25.1–36.8) | 23.2 (21.0–25.3) | 21.3 (18.9–23.7) |
| Psychological well-being |  | 23.2 (18.4–28.0) | 16.1 (14.5–17.8) | 16.5 (14.3–18.6) |
| Leisure activity |  | 38.8 (33.4–44.2) | 36.9 (34.6–39.2) | 40.7 (38.1–43.4) |
|  |  |  |  |  |
|  |  |  |  |  |
|  |  | **Income** | | |
|  |  | **low** | **medium** | **high** |
|  |  | % (95% CI) | % (95% CI) | % (95% CI) |
| Family |  | 21.6 (18.0–25.2) | 20.2 (18.3–22.1) | 15.3 (12.8–17.9) |
| Partnership |  | 11.3 (8.2–14.4) | 11.4 (9.8–13.0) | 7.2 (5.4–8.9) |
| Own financial situation |  | 21.8 (18.1–25.5) | 8.3 (6.8–9.8) | 2.8 (1.9–3.8) |
| Social life |  | 35.6 (31.3–39.8) | 32.7 (30.6–34.9) | 31.3 (27.9–34.8) |
| Work/ school |  | 29.8 (25.0–34.6) | 22.9 (21.0–24.9) | 20.6 (17.5–23.6) |
| Psychological well-being |  | 22.4 (18.7–26.1) | 16.9 (15.2–18.6) | 12.7 (10.3–15.1) |
| Leisure activity |  | 37.1 (32.7–41.4) | 39.5 (37.3–41.7) | 39.0 (35.5–42.4) |


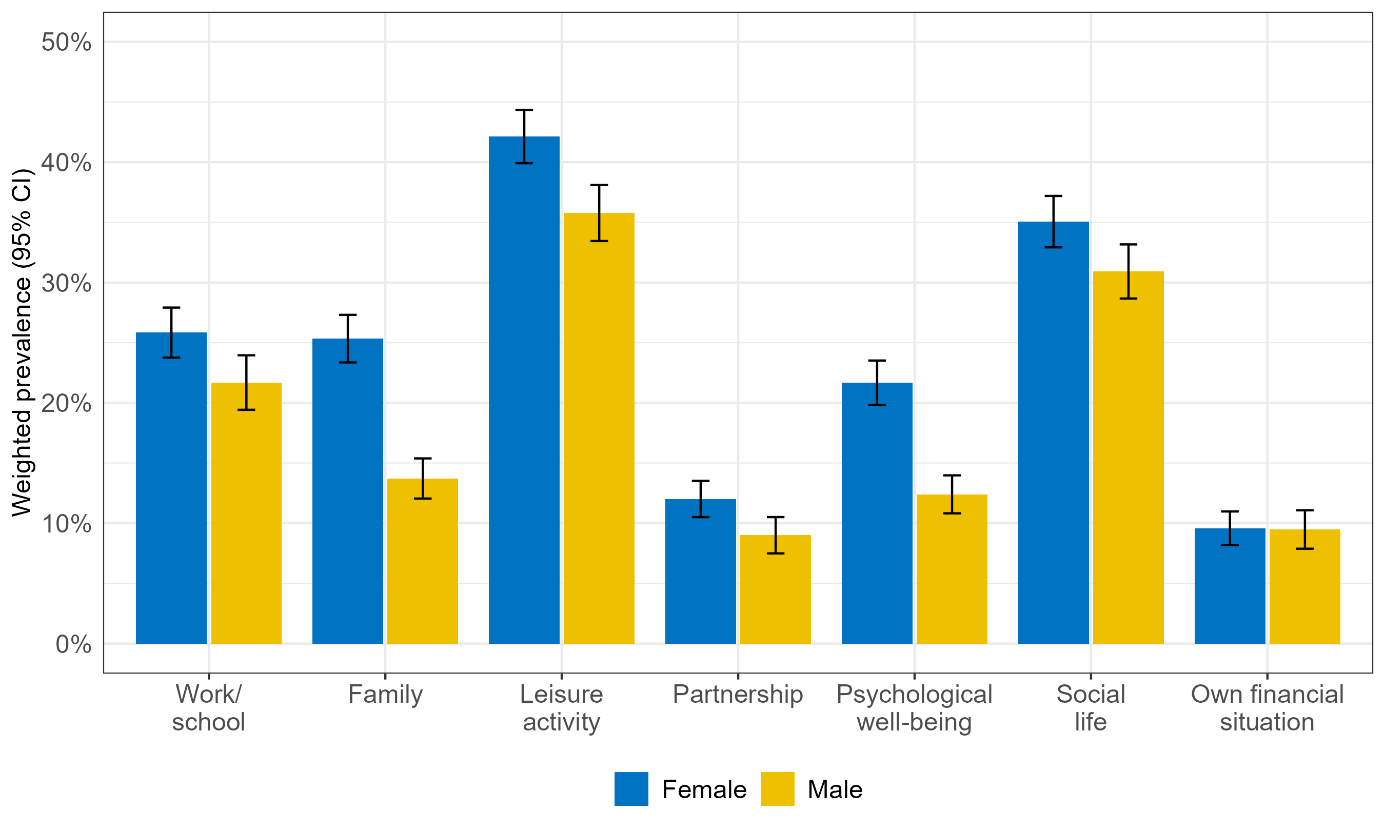


**Figure supp. 1** Life domain-specific prevalence estimates of pandemic-induced psychosocial stress (highly stressed) by sex

**
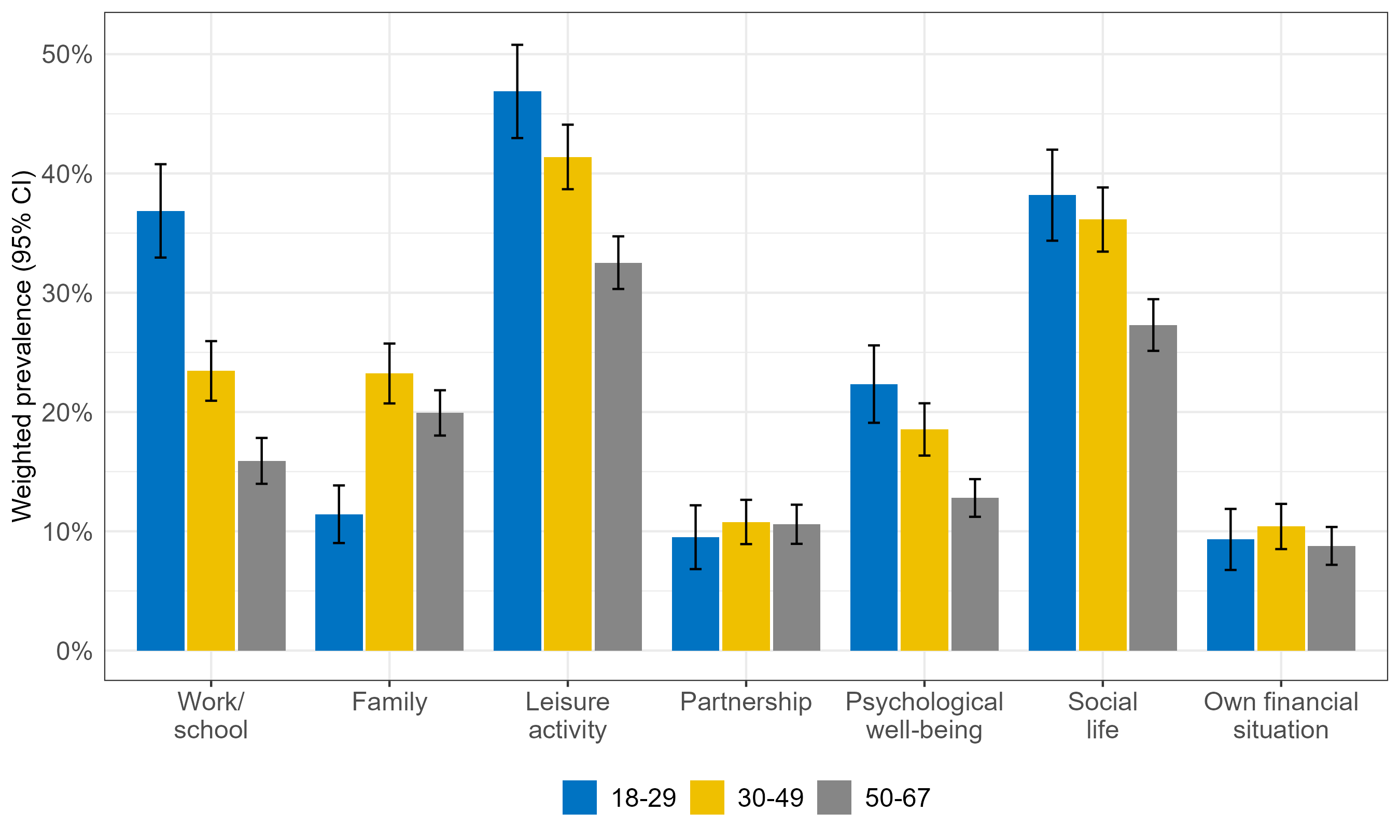
**

**Figure supp. 2** Life domain-specific prevalence estimates of pandemic-induced psychosocial stress (highly stressed) by age

**Table supp. 3** Adjusted prevalence ratios and 95%-confidence intervals (95% CI) for high pandemic-induced psychosocial stress by education and income (with high education and income as reference categories)

|  |  | **Education** | | | | |  | **Income** | | | | |
| --- | --- | --- | --- | --- | --- | --- | --- | --- | --- | --- | --- | --- |
|  |  | **low** | |  | **medium** | |  | **low** | |  | **medium** | |
|  |  | PR | p |  | PR | p |  | PR | p |  | PR | p |
|  |  | 95% CI |  |  | 95% CI |  |  | 95% CI |  |  | 95% CI |  |
| Family |  | 1.2 | 0.179 |  | 0.92 | 0.298 |  | **1.35** | **0.029** |  | 1.21 | 0.063 |
|  |  | (0.92–1.57) |  |  | (0.80–1.07) |  |  | **(1.03–1.77)** |  |  | (0.99–1.49) |  |
|  |  |  |  |  |  |  |  |  |  |  |  |  |
| Partnership |  | **1.68** | **0.011** |  | 1.14 | 0.289 |  | 1.3 | 0.240 |  | **1.5** | **0.009** |
|  |  | **(1.13–2.49)** |  |  | (0.90–1.44) |  |  | (0.84–2.00) |  |  | **(1.11–2.03)** |  |
|  |  |  |  |  |  |  |  |  |  |  |  |  |
| Own financial situation |  | **2.43** | **<0.001** |  | **1.59** | **0.003** |  | **5.54** | **<0.001** |  | **2.41** | **<0.001** |
|  |  | **(1.53–3.86)** |  |  | **(1.17–2.16)** |  |  | **(3.61–8.52)** |  |  | **(1.64–3.53)** |  |
|  |  |  |  |  |  |  |  |  |  |  |  |  |
| Social life |  | 0.94 | 0.558 |  | **0.87** | **0.010** |  | **1.23** | **0.027** |  | 1.09 | 0.191 |
|  |  | (0.87–1.15) |  |  | **(0.78–0.97)** |  |  | **(1.02–1.48)** |  |  | (0.96–1.25) |  |
|  |  |  |  |  |  |  |  |  |  |  |  |  |
| Work/school |  | 1.01 | 0.954 |  | 0.91 | 0.307 |  | 1.33 | 0.071 |  | 1.11 | 0.317 |
|  |  | (0.70–1.45) |  |  | (0.77–1.09) |  |  | (0.98–1.80) |  |  | (0.91–1.35) |  |
|  |  |  |  |  |  |  |  |  |  |  |  |  |
| Psychological well-being |  | 1.21 | 0.184 |  | 0.93 | 0.406 |  | **1.52** | **0.005** |  | 1.24 | 0.058 |
|  |  | (0.91–1.60) |  |  | (0.79–1.10) |  |  | **(1.14–2.04)** |  |  | (0.99–1.55) |  |
|  |  |  |  |  |  |  |  |  |  |  |  |  |
| Leisure activity |  | 0.92 | 0.346 |  | 0.93 | 0.125 |  | 0.99 | 0.884 |  | 1.03 | 0.570 |
|  |  | (0.78–1.09) |  |  | (0.85–1.02) |  |  | (0.84–1.16) |  |  | (0.92–1.15) |  |
|  |  |  |  |  |  |  |  |  |  |  |  |  |

Adjusted for age, sex, employment status, frequency working from home and household composition. Statistically significant estimates and p-values in bold

**Table supp. 4a** Adjusted prevalence ratios and 95%-confidence intervals (95% CI) for high pandemic-induced psychosocial stress by education and income (with high education and income as reference categories) among women.

|  |  | **Education** | | | | |  | **Income** | | | | |
| --- | --- | --- | --- | --- | --- | --- | --- | --- | --- | --- | --- | --- |
|  |  | **low** | |  | **medium** | |  | **low** | |  | **medium** | |
|  |  | PR | p |  | PR | p |  | PR | p |  | PR | p |
|  |  | 95% CI |  |  | 95% CI |  |  | 95% CI |  |  | 95% CI |  |
| Family |  | 1.29 | 0.082 |  | 0.94 | 0.467 |  | **1.46** | **0.019** |  | **1.38** | **0.006** |
|  |  | (0.97–1.73) |  |  | (0.79–1.11) |  |  | **(1.07–2.01)** |  |  | **(1.10–1.75)** |  |
|  |  |  |  |  |  |  |  |  |  |  |  |  |
| Partnership |  | **1.62** | **0.047** |  | 1.08 | 0.603 |  | 1.46 | 0.179 |  | **1.56** | **0.024** |
|  |  | **(1.01–2.62)** |  |  | (0.80–1.47) |  |  | (0.84–2.51) |  |  | **(1.06–2.29)** |  |
|  |  |  |  |  |  |  |  |  |  |  |  |  |
| Own financial situation |  | **2.70** | **0.001** |  | **1.86** | **0.002** |  | **4.70** | **<0.001** |  | **2.02** | **0.012** |
|  |  | **(1.50–4.87)** |  |  | **(1.26–2.75)** |  |  | **(2.58–8.56)** |  |  | **(1.17–3.5)** |  |
|  |  |  |  |  |  |  |  |  |  |  |  |  |
| Social life |  | 0.90 | 0.412 |  | **0.81** | **0.003** |  | **1.25** | **0.047** |  | 1.07 | 0.422 |
|  |  | (0.71–1.15) |  |  | **(0.71–0.93)** |  |  | **(1.00–1.57)** |  |  | (0.90–1.27) |  |
|  |  |  |  |  |  |  |  |  |  |  |  |  |
| Work/school |  | 0.87 | 0.383 |  | 1.03 | 0.780 |  | 1.04 | 0.784 |  | 0.84 | 0.095 |
|  |  | (0.63–1.19) |  |  | (0.85–1.25) |  |  | (0.79–1.37) |  |  | (0.69–1.03) |  |
|  |  |  |  |  |  |  |  |  |  |  |  |  |
| Psychological well-being |  | 1.33 | 0.075 |  | 0.95 | 0.594 |  | **1.46** | **0.019** |  | 1.20 | 0.155 |
|  |  | (0.97–1.82) |  |  | (0.78–1.15) |  |  | **(1.06–1.99)** |  |  | (0.93–1.56) |  |
|  |  |  |  |  |  |  |  |  |  |  |  |  |
| Leisure activity |  | 0.91 | 0.391 |  | **0.86** | **0.012** |  | 1.06 | 0.546 |  | 1.05 | 0.499 |
|  |  | (0.73–1.13) |  |  | **(0.76–0.97)** |  |  | (0.87–1.29) |  |  | (0.91–1.20) |  |
|  |  |  |  |  |  |  |  |  |  |  |  |  |

Adjusted for age, employment status, frequency working from home and household composition. Statistically significant estimates and p-values in bold

**Table supp. 4b** Adjusted prevalence ratios and 95%-confidence intervals (95% CI) for high pandemic-induced psychosocial stress by education and income (with high education and income as reference categories) among men.

|  |  | **Education** | | | | |  | **Income** | | | | |
| --- | --- | --- | --- | --- | --- | --- | --- | --- | --- | --- | --- | --- |
|  |  | **low** | |  | **medium** | |  | **low** | |  | **medium** | |
|  |  | PR | p |  | PR | p |  | PR | p |  | PR | p |
|  |  | 95% CI |  |  | 95% CI |  |  | 95% CI |  |  | 95% CI |  |
| Family |  | 1.01 | 0.958 |  | 0.91 | 0.456 |  | 1.20 | 0.445 |  | 0.98 | 0.890 |
|  |  | (0.60–1.71) |  |  | (0.70–1.18) |  |  | (0.76–1.89) |  |  | (0.70–1.37) |  |
|  |  |  |  |  |  |  |  |  |  |  |  |  |
| Partnership |  | 1.66 | 0.130 |  | 1.20 | 0.334 |  | 1.12 | 0.737 |  | 1.42 | 0.125 |
|  |  | (0.86–3.18) |  |  | (0.83–1.72) |  |  | (0.58–2.14) |  |  | (0.91–2.22) |  |
|  |  |  |  |  |  |  |  |  |  |  |  |  |
| Own financial situation |  | **2.19** | **0.017** |  | 1.33 | 0.203 |  | **6.35** | **<0.001** |  | **2.80** | **<0.001** |
|  |  | **(1.15–4.17)** |  |  | (0.86–2.05) |  |  | **(3.55–11.35)** |  |  | **(1.73–4.53)** |  |
|  |  |  |  |  |  |  |  |  |  |  |  |  |
| Social life |  | 0.98 | 0.898 |  | 0.95 | 0.581 |  | 1.21 | 0.179 |  | 1.13 | 0.225 |
|  |  | (0.72–1.34) |  |  | (0.81–1.13) |  |  | (0.92–1.60) |  |  | (0.93–1.37) |  |
|  |  |  |  |  |  |  |  |  |  |  |  |  |
| Work/school |  | 1.13 | 0.545 |  | 0.88 | 0.303 |  | 1.39 | 0.105 |  | 1.36 | 0.036 |
|  |  | (0.76–1.68) |  |  | (0.69–1.12) |  |  | (0.93–2.08) |  |  | (1.02–1.82) |  |
|  |  |  |  |  |  |  |  |  |  |  |  |  |
| Psychological well-being |  | 0.95 | 0.846 |  | 0.94 | 0.686 |  | 1.60 | 0.079 |  | 1.31 | 0.159 |
|  |  | (0.56–1.62) |  |  | (0.72–1.24) |  |  | (0.95–2.71) |  |  | (0.90–1.92) |  |
|  |  |  |  |  |  |  |  |  |  |  |  |  |
| Leisure activity |  | 0.92 | 0.528 |  | 1.02 | 0.821 |  | 0.89 | 0.371 |  | 1.01 | 0.936 |
|  |  | (0.70–1.20) |  |  | (0.88–1.18) |  |  | (0.68–1.15) |  |  | (0.85–1.19) |  |
|  |  |  |  |  |  |  |  |  |  |  |  |  |

Adjusted for age, employment status, frequency working from home and household composition. Statistically significant estimates and p-values in bold

**Table supp. 5** Adjusted prevalence ratios and 95%-confidence intervals (95% CI) for high pandemic-induced psychosocial stress by education and income (with high education and income as reference categories) with "does not apply" operationalized as not stressed.

|  |  | **Education** | | | | |  | **Income** | | | | |
| --- | --- | --- | --- | --- | --- | --- | --- | --- | --- | --- | --- | --- |
|  |  | **low** | |  | **medium** | |  | **low** | |  | **medium** | |
|  |  | PR | p |  | PR | p |  | PR | p |  | PR | p |
|  |  | 95% CI |  |  | 95% CI |  |  | 95% CI |  |  | 95% CI |  |
| Family |  | 1.2 | 0.177 |  | 0.92 | 0.298 |  | **1.34** | **0.035** |  | 1.21 | 0.068 |
|  |  | (0.92–1.57) |  |  | (0.80–1.07) |  |  | **(1.02–1.76)** |  |  | (0.99–1.49) |  |
|  |  |  |  |  |  |  |  |  |  |  |  |  |
| Partnership |  | **1.57** | **0.033** |  | 1.11 | 0.372 |  | 1.28 | 0.267 |  | **1.46** | **0.016** |
|  |  | **(1.04–2.39)** |  |  | (0.88–1.41) |  |  | (0.83–1.97) |  |  | **(1.07–1.99)** |  |
|  |  |  |  |  |  |  |  |  |  |  |  |  |
| Own financial situation |  | **2.26** | **0.001** |  | **1.59** | **0.003** |  | **5.52** | **<0.001** |  | **2.39** | **<0.001** |
|  |  | **(1.41–3.62)** |  |  | **(1.17–2.16)** |  |  | **(3.60–8.57)** |  |  | **(1.64–3.50)** |  |
|  |  |  |  |  |  |  |  |  |  |  |  |  |
| Social life |  | 0.93 | 0.459 |  | **0.87** | **0.008** |  | **1.21** | **0.039** |  | 1.09 | 0.217 |
|  |  | (0.77–1.13) |  |  | **(0.78–0.96)** |  |  | **(1.01–1.46)** |  |  | (0.95–1.25) |  |
|  |  |  |  |  |  |  |  |  |  |  |  |  |
| Work/school |  | 0.96 | 0.788 |  | 0.93 | 0.365 |  | 1.27 | 0.103 |  | 1.08 | 0.406 |
|  |  | (0.69–1.32) |  |  | (0.78–1.09) |  |  | (0.95–1.70) |  |  | (0.90–1.31) |  |
|  |  |  |  |  |  |  |  |  |  |  |  |  |
| Psychological well-being |  | 1.17 | 0.261 |  | 0.93 | 0.372 |  | **1.51** | **0.006** |  | 1.23 | 0.067 |
|  |  | (0.89–1.55) |  |  | (0.79–1.09) |  |  | **(1.12–2.03)** |  |  | (0.99–1.54) |  |
|  |  |  |  |  |  |  |  |  |  |  |  |  |
| Leisure activity |  | 0.89 | 0.188 |  | 0.93 | 0.117 |  | 0.96 | 0.647 |  | 1.03 | 0.658 |
|  |  | (0.75–1.06) |  |  | (0.84–1.02) |  |  | (0.82–1.14) |  |  | (0.92–1.14) |  |
|  |  |  |  |  |  |  |  |  |  |  |  |  |

Adjusted for age, sex, employment status, frequency working from home and household composition. Statistically significant estimates and p-values in bold

**Table supp. 6** Adjusted prevalence ratios and 95%-confidence intervals (95% CI) for pandemic-induced psychosocial stress (“rather stressed”/”highly stressed”) by education and income (with high education and income as reference categories)

|  |  | **Education** | | | | |  | **Income** | | | | |
| --- | --- | --- | --- | --- | --- | --- | --- | --- | --- | --- | --- | --- |
|  |  | **low** | |  | **medium** | |  | **low** | |  | **medium** | |
|  |  | PR | p |  | PR | p |  | PR | p |  | PR | p |
|  |  | 95% CI |  |  | 95% CI |  |  | 95% CI |  |  | 95% CI |  |
| Family |  | 0.96 | 0.527 |  | 0.94 | 0.089 |  | 1.10 | 0.137 |  | **1.15** | **0.003** |
|  |  | (0.84–1.09) |  |  | (0.88–1.01) |  |  | (0.97–1.26) |  |  | **(1.05–1.26)** |  |
|  |  |  |  |  |  |  |  |  |  |  |  |  |
| Partnership |  | 1.15 | 0.204 |  | 1.00 | 0.985 |  | 1.19 | 0.149 |  | **1.35** | **<0.001** |
|  |  | (0.93–1.43) |  |  | (0.88–1.13) |  |  | (0.94–1.52) |  |  | **(1.15–1.59)** |  |
|  |  |  |  |  |  |  |  |  |  |  |  |  |
| Own financial situation |  | **1.92** | **<0.001** |  | **1.42** | **<0.001** |  | **3.11** | **<0.001** |  | **1.87** | **<0.001** |
|  |  | **(1.54–2.39)** |  |  | **(1.21–1.65)** |  |  | **(2.39–4.05)** |  |  | **(1.48–2.36)** |  |
|  |  |  |  |  |  |  |  |  |  |  |  |  |
| Social life |  | **0.91** | **0.026** |  | **0.93** | **0.001** |  | 1.02 | 0.622 |  | 1.03 | 0.204 |
|  |  | **(0.84–0.99)** |  |  | **(0.89–0.97)** |  |  | (0.94–1.10) |  |  | (0.98–1.09) |  |
|  |  |  |  |  |  |  |  |  |  |  |  |  |
| Work/school |  | 0.95 | 0.609 |  | 0.93 | 0.108 |  | 1.15 | 0.080 |  | 1.05 | 0.337 |
|  |  | (0.79–1.15) |  |  | (0.84–1.02) |  |  | (0.98–1.34) |  |  | (0.95–1.18) |  |
|  |  |  |  |  |  |  |  |  |  |  |  |  |
| Psychological well-being |  | 1.04 | 0.532 |  | 0.93 | 0.063 |  | **1.17** | **0.017** |  | **1.16** | **0.003** |
|  |  | (0.92–1.18) |  |  | (0.87–1.00) |  |  | **(1.03–1.33)** |  |  | **(1.05–1.27)** |  |
|  |  |  |  |  |  |  |  |  |  |  |  |  |
| Leisure activity |  | **0.91** | **0.027** |  | **0.94** | **0.006** |  | **0.90** | **0.011** |  | 0.98 | 0.355 |
|  |  | **(0.83–0.99)** |  |  | **(0.90–0.98)** |  |  | **(0.83–0.98)** |  |  | (0.93–1.03) |  |
|  |  |  |  |  |  |  |  |  |  |  |  |  |

Adjusted for age, sex, employment status, frequency working from home and household composition. Statistically significant estimates and p-values in bold
